# Notch1 Mediates Preconditioning Protection Induced by GPER in Normotensive and Hypertensive Female Rat Hearts

**DOI:** 10.3389/fphys.2018.00521

**Published:** 2018-05-15

**Authors:** Carmine Rocca, Saveria Femminò, Giorgio Aquila, Maria C. Granieri, Ernestina M. De Francesco, Teresa Pasqua, Damiano C. Rigiracciolo, Francesca Fortini, Maria C. Cerra, Marcello Maggiolini, Pasquale Pagliaro, Paola Rizzo, Tommaso Angelone, Claudia Penna

**Affiliations:** ^1^Laboratory of Molecular and Cellular Cardiac Physiology, Department of Biology, Ecology and E.S., University of Calabria, Rende, Italy; ^2^Department of Biological and Clinical Sciences, University of Turin, Turin, Italy; ^3^Department of Medical Sciences, University of Ferrara, Ferrara, Italy; ^4^Department of Pharmacy, Health and Nutritional Sciences, University of Calabria, Rende, Italy; ^5^Maria Cecilia Hospital, GVM Care & Research, E.S. Health Science Foundation, Cotignola, Italy; ^6^National Institute for Cardiovascular Research, Bologna, Italy; ^7^Department of Morphology, Surgery and Experimental Medicine, University of Ferrara, Ferrara, Italy; ^8^Laboratory for Technologies of Advanced Therapies, University of Ferrara, Ferrara, Italy

**Keywords:** cardioprotection, preconditioning, H9c2, isolated rat hearts, reperfusion injury salvage kinases, PI3K/Akt, NOS

## Abstract

G protein-coupled estrogen receptor (GPER) is an estrogen receptor expressed in the cardiovascular system. G1, a selective GPER ligand, exerts cardiovascular effects through activation of the PI3K-Akt pathway and Notch signaling in normotensive animals. Here, we investigated whether the G1/GPER interaction is involved in the limitation of infarct size, and improvement of post-ischemic contractile function in female spontaneous hypertensive rat (SHR) hearts. In this model, we also studied Notch signaling and key components of survival pathway, namely PI3K-Akt, nitric oxide synthase (NOS) and mitochondrial K^+^-ATP (MitoKATP) channels. Rat hearts isolated from female SHR underwent 30 min of global, normothermic ischemia and 120 min of reperfusion. G1 (10 nM) alone or specific inhibitors of GPER, PI3K/NOS and MitoKATP channels co-infused with G1, just before I/R, were studied. The involvement of Notch1 was studied by Western blotting. Infarct size and left ventricular pressure were measured. To confirm endothelial-independent G1-induced protection by Notch signaling, H9c2 cells were studied with specific inhibitor, *N*-[*N*-(3,5 difluorophenacetyl)-L-alanyl]-*S*-phenylglycine *t*-butyl ester (DAPT, 5 μM), of this signaling. Using DAPT, we confirmed the involvement of G1/Notch signaling in limiting infarct size in heart of normotensive animals. In the hypertensive model, G1-induced reduction in infarct size and improvement of cardiac function were prevented by the inhibition of GPER, PI3K/NOS, and MitoKATP channels. The involvement of Notch was confirmed by western blot in the hypertensive model and by the specific inhibitor in the normotensive model and cardiac cell line. Our results suggest that GPERs play a pivotal role in mediating preconditioning cardioprotection in normotensive and hypertensive conditions. The G1-induced protection involves Notch1 and is able to activate the survival pathway in the presence of comorbidity. Several pathological conditions, including hypertension, reduce the efficacy of ischemic conditioning strategies. However, G1-induced protection can result in significant reduction of I/R injury also female in hypertensive animals. Further studies may ascertain the clinical translation of the present results.

## Introduction

The G protein-coupled estrogen receptor (GPR30/GPER) is expressed in the heart and it mediates non-genomic effects of estrogen. It has been suggested that GPER activation mediates beneficial effects in the cardiovascular system, as demonstrated using pharmacological agonists/antagonists of GPER (Dennis et al., 2009). In this regard, the synthetic and selective GPER agonist, G1 (Bologa et al., 2006), has been shown to activate signaling pathways involved in cardiomyocytes survival, thus improving cardiovascular function both in normal and stressful conditions. Yet, the synthetic and selective GPER antagonist, G15 (Dennis et al., 2009), prevented these beneficial effects (De Francesco et al., 2013, 2017). To obtain its effect, GPER cross-reacts with a number of cell signaling systems, including the epidermal growth factor receptor, the mitogen-activated protein kinases and the Notch signaling pathway (Pupo et al., 2016).

In the normotensive rat, GPER activation improves contractile recovery and limits infarct size in isolated rat hearts following ischemia/reperfusion (I/R) through a gender-independent and PI3K-dependent mechanism (Deschamps and Murphy, 2009).

The potential of ischemic preconditioning to reduce I/R injury has been recognized more than 30 years ago (Hausenloy et al., 2016). Preconditioning limits I/R injury *via* multiple pathways. However, the effectiveness of this cardioprotective intervention is noticeably reduced in pathological animal models, such as hypertensive animals (Ferdinandy et al., 2007).

Several data demonstrated that in both normotensive male and female rodent models, the GPER activation plays a role as pre- and post-conditioning cardioprotective agent *in vitro* and *ex vivo* (Deschamps and Murphy, 2009; Bopassa et al., 2010; Deschamps et al., 2010; Li et al., 2015; Feng et al., 2017; Menazza et al., 2017). These GPER-dependent cardioprotective effects are displayed by its ability to improve the functional recovery, to preserve the mitochondrial structural integrity and function and to reduce mitophagy.

However, the potential of GPER to mediate beneficial effects in hypertensive conditions has not yet been fully investigated.

In male spontaneously hypertensive rats (SHRs) hearts, we have reported that the activation of GPER reduced the expression of apoptotic and fibrotic factors and induced negative inotropic and lusitropic effects (De Francesco et al., 2013). In these hearts, GPER induced activation Akt/PKB, ERK1/2, GSK-3β, c-Jun and endothelial nitric oxide (NO) synthase (eNOS) signaling. Also, GPER prevents the detrimental cardiac effects of certain anti-cancer agents like Doxorubicin (De Francesco et al., 2017). Hence, GPER may represent a novel pharmacological target in the treatment of some cardiovascular pathologies associated with stressful conditions, such as hypertension (De Francesco et al., 2013).

Besides cross-talking with GPER in breast cancer cell lines (Pupo et al., 2014), Notch signaling pathway plays an important role in regulating cell death, differentiation, and angiogenesis (Lubecka et al., 2016). Moreover, it is associated with cardioprotection. Indeed, Notch signaling pathway activation reduces I/R injury and modulates cardiac repair after myocardial infarction (Li et al., 2010). Importantly, Notch drives cell survival signaling contributing to cardioprotection by ischemic conditioning protocols in healthy animals (Zhou et al., 2013). Therefore, we hypothesized that GPER/Notch pathway may be involved in the cardioprotection mediated by GPER-agonist in hypertensive female models. To ascertain this hypothesis, we studied GPER/Notch pathway in the heart of hypertensive model, firstly using the two synthetic molecules G1 and G15, which act as selective and potent agonist and antagonist of GPER, respectively. These allowed to discriminate the selective GPER activation from the estrogen effects mediated by the classical intracellular estrogen receptors (ER α/β). In addition, in order to further explore the mechanism of action GPER-dependent, specific inhibitors of PI3K/NOS pathway and mitoKATP channels were used. The Notch involvement was studied by Western blot analysis. For comparative purpose, we confirmed the role of GPER/Notch pathway in hearts of normotensive animals using specif inhibitors of GPER and Notch pathway. To further analyze a direct cardioprotective effect of GPER agonist, we studied its effect in an *in vitro* model of injury: we subjected rat embryonic-heart derived cardiomyoblasts (H9c2) to hypoxia/reoxygenation with and without inhibitors of PI3K/NOS pathway and MitoKATP channels.

## Materials and Methods

### Animals

Female normotensive Wistar rats (*n* = 25; body weight: 250–300 g; Harlan Laboratories, Udine, Italy) and Female SHRs (*n* = 28; body weight: 250–300; Harlan Laboratories, Udine, Italy) received humane care in compliance with the Guide for the Care and Use of Laboratory Animals published by the United States National Institutes of Health (NIH Publication No. 85-23, revised 1996). Two normotensive Wistar rat hearts and one SHR heart were discarded due to their very low left ventricular developed pressure or other technical issues after connection to the perfusion line. In accordance with the Italian law in force (DL n. 116, January 27, 1992), regarding animal protection, the scientific project has been approved by the Italian Ministry of Health (Rome, Italy). All animals were identically housed under controlled light and temperature conditions with access to food and water *ad libitum*. SHR female rats, used in this paper for hemodynamic studies, are part of a set of hypertensive animals whose male counterpart was employed in previous works (De Francesco et al., 2013; Pasqua et al., 2015). The basal blood pressure of female animals (recorded before experiments by the tail-cuff method) displayed the same trend of male rats (data not shown).

### Experimental Models

#### Isolated Perfused Heart

The methods were similar to those previously described (Penna et al., 2009, 2011; Pasqua et al., 2015). Briefly, each animal was heparinized (800 U/100 g b.w., i.m.) and after 10 min rats were anesthetized with i.p. injection of ethyl carbamate (2 g/kg rat), then hearts were rapidly excised and immediately arrested in ice-cold buffered Krebs–Henseleit solution (KHS) for subsequent aorta cannulation. Hearts were then perfused at constant retrograde flow (12 ml/min) and constant temperature of 37°C. Perfusion medium was a modified Krebs–Henseleit Solution (KHS; pH 7.4) gassed with 95% O_2_ and 5% CO_2_ containing (in mM): 113.0 NaCl; 4.7 KCl; 1.2 MgSO_4_; 25.0 NaHCO_3_; 1.2 KH_2_PO_4_; 1.8 CaCl_2_; 11.0 glucose; 1.1 mannitol; 5.0 Na-pyruvate (Pasqua et al., 2015). A water-filled latex balloon was connected to a pressure transducer (BLPR, WRI, Inc., Saratota, FL, United States) and pushed into the left ventricle (LV) through the mitral valve. Another pressure transducer connected with perfusion cannula was used to measure coronary pressure (CP). The developed left ventricular pressure (dLVP; mmHg, index of contractile activity) and the left ventricular end diastolic pressure (LVEDP; mmHg, index of contracture) were measured to evaluate cardiac function. The LVEDP was set to obtain a 5–8 mmHg pressure. Cardiac performance was recorded by using the PowerLab data acquisition system. Parameters were quantified by using Chart Software (ADInstruments, Oxford, United Kingdom) (Pasqua et al., 2015).

**FIGURE 1:**
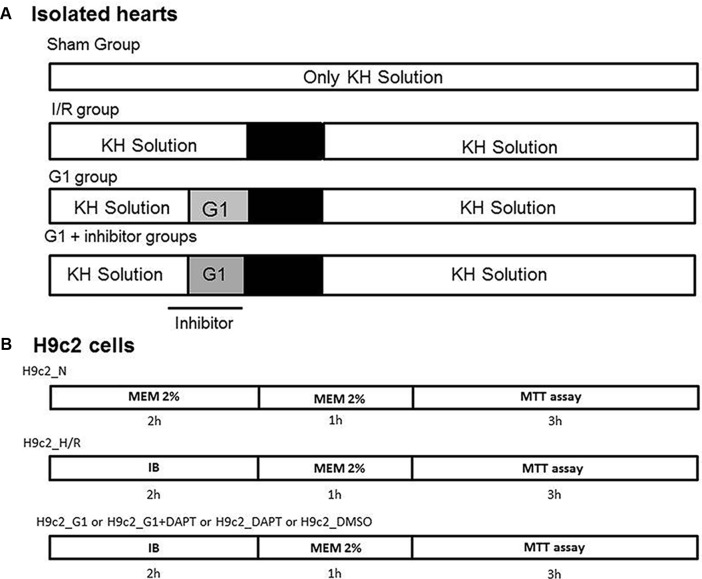
Timeline of experimental protocols. **(A)** Protocols of ischemia/reperfusion (I/R) in isolated rat hearts and Sham group. G1 was given for 20 min before ischemia and inhibitors (DAPT, inhibitor of Notch or G15, selective GPER antagonist or Wortmannin, a specific inhibitor of PI3K activity or L-N5-(1-iminoethyl)ornithine (L-NIO), a specific inhibitor of nitric oxide synthase (NOS), or 5-hydroxydecanoate (5HD), a specific inhibitor of mitochondrial ATP-sensitive potassium [MitoK(ATP)]) were given 5 min before G1 and was stopped at the end of the 20 min of infusion of G1. **(B)** Protocols of normoxia and hypoxia/reoxygenation (H/R) in H9c2 cells. G1 or G1+DAPT or DMSO were given before hypoxia/reoxygenation protocol. MTT: 3-(4,5-dimethylthiazol-2-yl)-2,5-diphenyltetrazolium bromide dye is used as colorimetric assay for assessing cell metabolic activity, reflecting the number of viable cells present. IB, ischemic buffer.

After stabilization, reference parameters were recorded and each heart was assigned in random order to one of the following experimental groups described below (**Figure 1A**).

#### Normotensive Heart Model

Four groups of hearts isolated from normotensive animals were subjected to the following protocols:

(1) Sham group (*n* = 3): hearts were stabilized for 30 min and then subjected to 150 min of perfusion only.
(2) I/R group (*n* = 8): after a 30 min stabilization period, the perfusion was completely stopped (global no-flow ischemia) for a duration of 30 min. Thereafter, hearts were reperfused for 120 min.
(3) GPER agonist G1 (G1) group (*n* = 7): hearts were pre-treated with G1 (10 nM), a GPER selective agonist for 20 min. Thereafter the hearts were subjected to I/R protocol, as above (De Francesco et al., 2013).
(4) G1+Notch inhibitor group (*n* = 5): hearts were pre-treated with a co-infusion of G1 and a selective inhibitor of Notch pathway, namely *N*-[*N*-(3,5 difluorophenacetyl)-L-alanyl]-*S*-phenylglycine *t*-butyl ester (DAPT, 5 μM) (Boccalini et al., 2015), and then four hearts were subjected to I/R.

#### Hypertensive Heart Model

In the following groups: Sham (*n* = 3), I/R (*n* = 3), G1 (*n* = 5), hearts isolated from female SHR were subjected to the protocols described above for normotensive animals.

Moreover, to deepen the signaling pathway involved in protection, the following groups were studied in the hypertensive model:

(8) G1+G15 group (*n* = 4): hearts were pre-treated with a co-infusion of the selective GPER agonist, G1 (10 nM), and the selective GPER antagonist G15 (100 nM) (De Francesco et al., 2013) and then subjected to I/R.
(9) G1+ WT group (*n* = 4): after stabilization, hearts were pretreated with a co-infusion of G1 (10 nM) and WT (100 nM) (Deschamps and Murphy, 2009; Penna et al., 2017).
(10) G1+L-NIO group (*n* = 4): after stabilization, hearts were pretreated with a co-infusion of G1 (10 nM) and L-NIO (10 μM) (El-Mas et al., 2009; De Francesco et al., 2013).
(11) G1+5HD group (*n* = 4): after stabilization, hearts were pretreated with a co-infusion of G1 (10 nM) and 5HD (100 μM) (Ajmani et al., 2011; Perrelli et al., 2013).

G1 concentration was selected on the basis of previous experiments (De Francesco et al., 2013).

The concentration for each pharmacological inhibitor was selected on the basis of preliminary dose–response curves, and according to the literature, as the first dose that did not significantly affect cardiac performance (De Francesco et al., 2013; Perrelli et al., 2013; Boccalini et al., 2015; Penna et al., 2017). The stability of the preparations was previously assessed by measuring each variable considered every 10 min. The stability of the preparation was on average 180 min.

In line with 3R principle for more ethical use of animals, we used less than four animals in those groups in which we have previous evidence of similar results (Penna et al., 2009, 2010, 2012; Rocca et al., 2018).

#### Cell Culture

The cardiac myoblast H9c2 cell line (ATCC, CRL-1446) was maintained in DMEM supplemented with 10% heat-inactivated fetal bovine serum (FBS) and 1% (v/v) streptomycin/penicillin in a humidified incubator at 37°C under 5% CO_2_ air prior to use (Hescheler et al., 1991).

In all sets of experiments, 80% confluent flasks were detached, counted in the Burker chamber and plated in 96 wells plate at a density of 5000 cells/well. Cells were then left for 24-h in standard culture conditions before applying the hypoxia/reoxygenation (H/R) protocols.

#### Hypoxia/Reoxygenation (H/R) Protocol

In this set of experiments, performed for comparative purposes, cells were cultured in normoxic conditions (21% O_2_ and 5% CO_2_) or in conditions of Hypoxia/Reoxygenation (1% O_2_ and 5% CO_2_ for 2 h, and subsequently 21% O_2_ and 5% CO_2_ for 1 h).

As Normoxia (N) we considered cell survival studied during standard H9c2 culture conditions.

As Hypoxia/Reoxygenation (H/R) we considered cell survival studied with for 2-h and a subsequent reoxygenation for 1-h. H/R protocol was obtained in a hypoxic chamber (INVIVO2 200, Belsar, Varese, Italy) and with an “Ischemic Buffer” (IB) containing (in mM): 137 NaCl; 12 KCl; 0.49 MgCl_2_; 0.9 CaCl_2_; 4 HEPES; 20 sodium lactate (pH 6.2) (Yin et al., 2013).

Before hypoxia, cells were pre-treated with G1 (10 nM) (De Francesco et al., 2013), or G1+ DAPT, (5 μM); (Boccalini et al., 2015) dissolved in DMEM-2% FBS in normoxic conditions and in IB in hypoxic conditions. Experimental groups, recapitulated in **Figure 1B**, are as follows:

(a) Control Groups, untreated cells cultured in normoxic conditions (H9c2-N); untreated cells subjected to H/R (H9c2-H/R).
(b) G1 Group, cells pre-treated with G1 (10 nM) and then subject to H/R protocol (H9c2_G1_H/R).
(c) G1+DAPT Group, cells pre-treated with G1 + DAPT (5 μM) and then subject to H/R protocol (H9c2_G1+DAPT_H/R).
(d) DAPT Group, cells pre-treated with DAPT and then subject to H/R protocol (H9c2_DAPT_H/R).
(e) DMSO Group, cells pre-treated with DMSO (0.1%) and then subject to H/R protocol (H9c2_DMSO_H/R).

#### Assessment of Myocardial Injury

Infarct mass was measured as usual in our laboratories (Penna et al., 2014; Pasqua et al., 2015). In short, the hearts were detached from the perfusion system at the end of the reperfusion and the left ventricles cut into circumferential sections about 2 mm thick. The heart slices were incubated for 20 min at 37°C in a 0.1% nitro-blue tetrazolium solution with phosphate buffer. The non-colored necrotic tissue was carefully separated from the vital tissue colored by an observer who was unaware of the studied protocol. The necrotic mass was gravimetrically weighed and expressed as a percentage of total left ventricular mass that was considered to be a risk area for global ischemia (Penna et al., 2014).

#### MTT Assay

At the end of all experiments, cell viability was assessed using the 3-(4,5-Dimethylthiazol-2-yl)-2,5-diphenyltetrazolium bromide (MTT) kit (Pasqua et al., 2015). The absorbance was measured at 570 nm using a microplate reader and the results were expressed as a percentage of control.

#### Western Blotting and Densitometric Analysis

Immunoblot procedures and analyses were conducted as previously described (Rocca et al., 2018). Immediately after the hearts had undergone the specific protocols described above (Sham, I/R, I/R+DAPT, I/R+G1, I/R+G1+DAPT for normotensive group and Sham, I/R, G1, and G1+G15 for hypertensive group), the cardiac apices were frozen in liquid nitrogen before being stored at -80°C until protein extraction (Penna et al., 2014). Myocardial tissues of the above groups were homogenized in a frozen RIPA lysis buffer (Sigma-Aldrich, St. Louis, MO, United States) containing a mixture of protease inhibitors (1 mM of aprotinin, 20 mM of phenylmethylsulfonyl fluoride, and 200 mM of sodium orthovanadate). Subsequently, myocardial homogenates were centrifuged at 15,000 × *g* for 25 min at 4°C for debris removal. Protein concentration was assessed using a Bradford reagent following the procedure described by the manufacturer (Sigma-Aldrich, St. Louis, MO, United States).

30 μg of total protein were separated on 10% SDS-PAGE gel [for β-actin, phospho-protein kinase B (p-Akt), total Akt (t-Akt), total Notch1, and cleaved Notch1)] or 8% Gel SDS-PAGE [for phospho-endothelial NOS (p-eNOS) and total eNOS (t-eNOS)], subjected to electrophoresis and transferred to polyvinyl-fluoride membranes (PVDFs). The non-specific binding was blocked by incubating the membranes with a buffered saline solution (TBS)/Tween 0.1%, pH 7.6 (TBST), containing 5% of fat-free dried milk and 0.5% BSA, for 1 h at room temperature. The PVDF membranes were incubated overnight at 4°C with a goat polyclonal antibody for Notch1 (C-20; Santa Cruz, CA, United States), which recognizes the carboxy-terminal of the Notch1 receptor, to detect the precursor of Notch1 (Notch1-PR, 250 kDa) (Caliceti et al., 2013; Fortini et al., 2017) or rabbit monoclonal antibody against cleaved Notch (Cell Signaling Technology, Inc., Danvers, MA, United States), which detects endogenous levels of intracellular Notch1 domain (NICD) when released from the cleavage between Gly1753 and Val1754. Samples were also incubated with rabbit polyclonal antibody against p-Akt, Akt, or eNOS (Santa Cruz Biotechnology, Inc., Dallas, TX, United States), with monoclonal goat antibody against p-eNOS (Santa Cruz Biotechnology, Inc., Dallas, TX, United States), with mouse monoclonal antibody against β-actin (Santa Cruz Biotechnology, Inc., Dallas, TX, United States), or with mouse monoclonal antibodies to GAPDH (Cell Signaling Technology, Inc., Danvers, TX, United States). The antibodies were diluted 1:1000 in TBST containing 5% BSA (TBSTM). Antibodies against Akt, eNOS, GAPDH, and β-actin were used as loading controls. After washing them with TBST three times, the membranes were incubated for about 1 h at room temperature with antibodies conjugated with secondary peroxidase (1:1000) in TBSTM. Immunodetection was done using the enhanced chemiluminescence kit ECL PLUS (GE Healthcare, Amersham, United Kingdom). Autoradiographs were obtained by exposing the membrane films to X-ray (Hyperfilm ECL, Amersham, United Kingdom). We then proceeded to digitize the immunoblots that were subjected to densitometric analysis of the bands. The analysis was performed by evaluating the areas and the intensity of the pixels represented by 256 values of Gray (0 = white; 256 = black). As usual, the background has been subtracted. The analyses were performed using NIH IMAGE 1.6 (National Institutes of Health, Bethesda, MD, United States).

#### Chemicals

1-[4-(-6-Bromobenzol[1,3]diodo-5-yl)-3a,4,5,9-btetrahydro-3H-cyclopenta[c-]quinolin8yl]ethanone (G-1) and (3aS,4R,9bR)-4-(6-bromo-1,3-benzodioxol-5-yl)-3a,4,5,9b-3H-cyclopenta[c] quinolone (G15) were from Tocris Bioscience, distributed by Space (Milan, Italy). Wortmannin (WT), a specific inhibitor of PI3K activity, L-N5-(1-iminoethyl)ornithine (L-NIO), a specific inhibitor of nitric oxide synthase (NOS), 5-hydroxydecanoate (5HD), a specific inhibitor of MitoK(ATP) channels, and DAPT, a γ-secretase complex inhibitor, were purchased from Sigma-Aldrich (Milan, Italy). Reagents were dissolved in dimethylsulfoxide (DMSO). Preliminary experiments showed that the presence of equivalent amounts of DMSO in Krebs–Henseleit solution (KHs) did not modify basal cardiac performance. All drug-containing solutions were freshly prepared just before the experiments.

### Statistical Analysis

All data were reported as mean ± SEM and analyzed by one-way analysis of variance (ANOVA). Non-parametric Newman–Keuls multiple comparison test (for post-ANOVA comparisons) was used for western blot and hemodynamic analyses. Differences at ^∗^*p* = < 0.05, ^∗∗^*p* = < 0.01, ^∗∗∗^*p* = < 0.001, ^∗∗∗∗^*p* = < 0.0001 were considered statistically significant. The statistical analyses were carried out using GraphPad Prism5.

## Results

The cardioprotective effects of G1 in isolated heart models were studied by comparing the effects elicited by I/R protocols with those induced by the GPER-agonist, G1, used as a pre-conditioning factor (PreC).

### Normotensive Rat Model

#### Cardioprotective Effect of GPER

##### Infarct size reduction by G1 preconditioning

**FIGURE 2:**
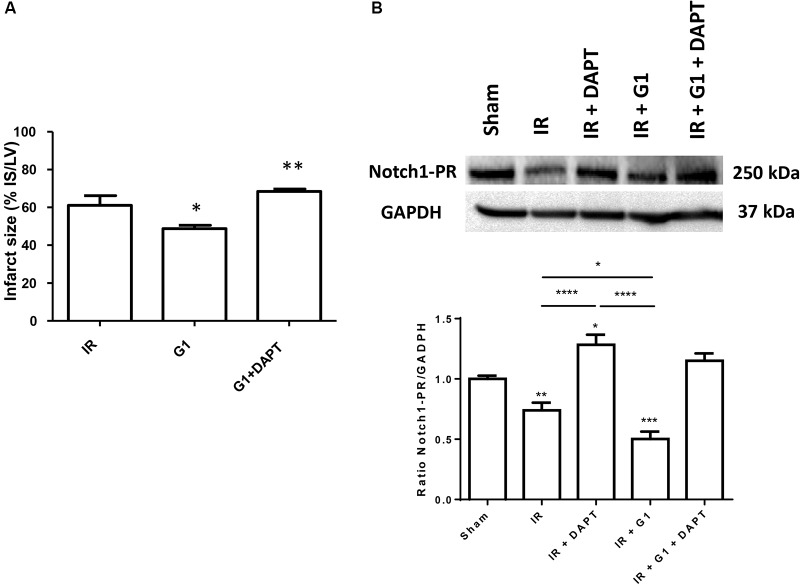
Cardioprotective effects of G1 in normotensive female rats were abolished by Notch1 inhibitor. **(A)** Infarct size. The amount of necrotic tissue measured after I/R protocols (30-min global ischemia and 120-min reperfusion) is reported as percent of the left ventricle mass (LV; % IS/LV) for I/R (*n* = 8), G1 (*n* = 7), and G1+DAPT groups (*n* = 4). **(B)** Western blotting (upper panel) of rat heart homogenates electrophoresed and immunoblotted with Notch1 C-20 antibody. GAPDH was used as loading control. Histograms (bottom panel) represent the ratio of densitometric analysis of protein/loading control normalized to Sham. The level of Notch1-PR was reduced in heart after I/R injury, compared to Sham group, indicating activation of Notch1, activation further increased by G1 treatment. DAPT treatment increased Notch1-PR protein level in heart subjected to I/R injury, both in the presence and absence of G1, suggestive of an inhibition of Notch1 activation. ^∗∗∗∗^*p* < 0.0001 IR vs. IR+DAPT and IR+G1 vs. IR+DAPT; ^∗∗∗^*p* < 0.001 IR+G1 vs. SHAM; ^∗∗^*p* < 0.01 IR vs. SHAM; ^∗^*p* < 0.05 IR+DAPT vs. SHAM and IR vs. IR+G1.

Here we confirmed the cardioprotective effect of GPER estrogen agonist, G1 (Bopassa et al., 2010; Li et al., 2015; Menazza et al., 2017). In particular, using an isolated rat heart model, infarct size was reduced from 63 ± 4% of risk area in I/R group to 48 ± 2% of risk area in G1 pretreated hearts (*p* < 0.01) (**Figure 2A**).

##### Involvement of Notch pathway in cardioprotective effect of GPER

The G1 cardioprotective effect was nullified by treatment with DAPT, an inhibitor of the γ-secretase, the enzyme required for the Notch1 cleavage and activation, co-infused with G1 (infarct size 68 ± 1%; *p* < 0.05 spect to G1 group; *p* = NS respect to I/R group) (**Figure 2A**). To confirm the involvement of Notch downstream of GPER during I/R, we performed Western blot analyses on heart homogenates from different treatment groups and showed that I/R induces the activation of Notch1, as indicated by the reduction of the precursor form (PR) of the receptor (**Figure 2B**). G1 treatment further induced Notch1 processing and this effect was blocked by DAPT (**Figure 2B**). The full unedited gel is showed in the Supplementary Figure 1.

### Hypertensive Rat Model

After we demonstrated the involvement of the GPER/Notch pathway in normotensive hearts, we focused on studying the G1 cardioprotective pathway in the hypertensive model in the detail.

#### Cardioprotective Effect of GPER

##### Infarct size is reduced by G1 preconditioning

**FIGURE 3:**
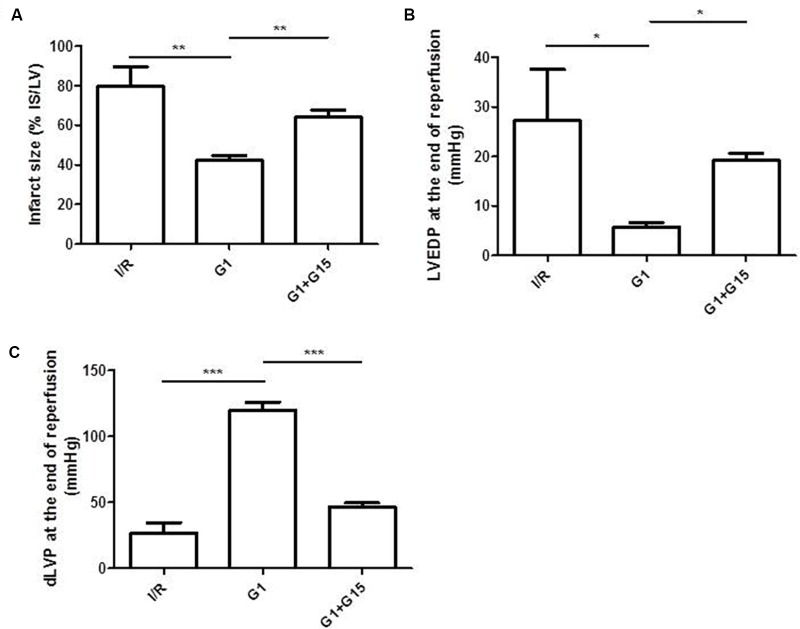
Cardioprotective effects of G1 in Hypertensive Female Rats (SHR) were abolished by GPER antagonistic specific inhibitor. **(A)** Infarct size. The amount of necrotic tissue measured after I/R protocols (30-min global ischemia and 120-min reperfusion) is reported as percentage of the left ventricle mass (LV; % IS/LV) for I/R (*n* = 3), G1 (*n* = 5), and G1+G15 groups (*n* = 4). **(B,C)** LVEDP and dLVP variations at the end of reperfusion. Data are expressed as changes of dLVP and LVEDP values (mmHg) at the end of the 120-min of reperfusion for I/R (*n* = 3), G1 (*n* = 5), and G1+G15 groups (*n* = 4). Changes were evaluated as mean ± SEM. Significant difference (one-way ANOVA, Newman–Keuls test): ^∗^*p* < 0.05, ^∗∗^*p* < 0.01, ^∗∗∗^*p* < 0.001.

Infarct size was reduced from 80 ± 9.6% of risk area in I/R group to 42 ± 2.5% of risk area in G1 pretreated hearts (*p* < 0.01) (**Figure 3A**). Moreover, when G1 was co-infused with the direct inhibitor of GPER, G15, hearts showed an infarct size similar to that found for I/R group (**Figure 3A**).

##### Post-ischemic diastolic and systolic functions are improved by G1 preconditioning

The effects mediated by G1 on diastolic and systolic functions were analyzed as previously described (De Francesco et al., 2013).

It is known that an increase in post-ischemic left ventricular end-diastolic pressure (LVEDP) of 4 mmHg or more above the pre-ischemic level indicates an important index of cardiac contracture. The 30 min ischemia and the subsequent reperfusion caused a sustained increase in LVEDP in I/R group (**Figure 3B**) (Pagliaro et al., 2003; Pasqua et al., 2015). Conversely, the preconditioning with G1 was able to abolish the contracture (**Figure 3B**), indicating that G1 significantly reduces the heart damage after I/R. Similar to the reduction in infarct size, the limitation of contracture was suppressed when G1 was co-infused with the direct inhibitor of GPER, G15.

Systolic function was evaluated by the level of dLVP recovery during reperfusion (i.e., inotropic index). SHR hearts from I/R group showed a limited dLVP recovery, while G1, administered before ischemia induction, significantly improved this function (**Figure 3C**). The co-infusion with the GPER-antagonist, G15, abolished the post-systolic recovery, indicating that G1 exerts a selective action on its receptor.

##### G1-induced infarct size limitation and improvement of post-ischemic contractile recovery is mediated by PI3K/NO/mKATP channels

**FIGURE 4:**
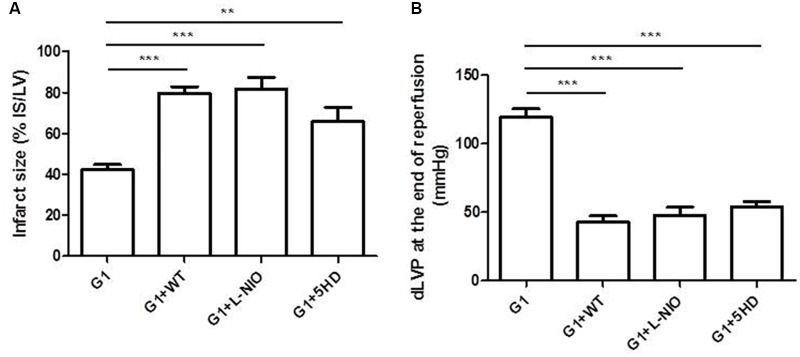
Cardioprotective effects of G1 in Hypertensive Female Rats (SHR) were abolished by specific inhibitors of PI3K/Akt-eNOS-MitoKATP channels. **(A)** Infarct size. The amount of necrotic tissue measured after I/R protocols (30-min global ischemia and 120-min reperfusion) is reported as percentage of the left ventricle mass (LV; % IS/LV) for G1 (*n* = 5) and G1+inhibitors (WT, L-NIO, and 5HD) groups (*n* = 4). **(B)** dLVP variations. Data are expressed as changes of dLVP values (mmHg) at the end of the 120-min of reperfusion for G1 (*n* = 5) and G1+inhibitors (WT, L-NIO, and 5HD) groups (*n* = 4). Changes were evaluated as mean ± SEM. Significant difference (one-way ANOVA, Newman–Keuls test): ^∗∗^*p* < 0.01, ^∗∗∗^*p* < 0.001.

In order to estimate the mechanism of action by which G1 exerted the cardioprotective effects in SHR hearts, the isolated and perfused hearts were co-treated with G1 plus specific inhibitors of survival signaling pathways involved in cardioprotection (i.e., WT to block PI3K, L-NIO to inhibit NOS, and 5HD to antagonize MitoKATP channels). **Figure 4** shows that all inhibitors abrogate the beneficial effects of G1 on all analyzed parameters. Indeed, G1-dependent reduction of infarct size was abolished in hearts co-treated with all the cardioprotective signaling antagonists (**Figure 4A**). Moreover, these inhibitors limited the post-ischemic systolic recovery elicited by G1, as demonstrated by the low values of dLVP reached at the end of reperfusion (**Figure 4B**). Similarly, the contracture limitation induced by G1 was suppressed in the presence of the above blockers (data not shown).

##### Effects of G1 as preconditioning agent on Notch and PI3K/Akt/NOS pathways

**FIGURE 5:**
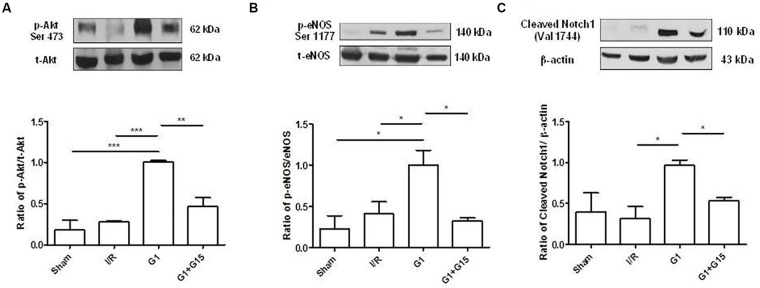
Effects of G1 given as preconditioning agent on Notch, PI3K/Akt/NOS Pathway Western Blot analysis of **(A)** p-Akt, **(B)** p-eNOS, and **(C)** cleaved Notch1 expression in cardiac extracts of Sham, I/R, G1, and G1+G15 groups (*n* = 3 hearts/group). Histograms represent the ratio of densitometric analysis of protein/loading control. Significant difference (one-way ANOVA, Newman–Keuls test): ^∗^*p* < 0.05, ^∗∗^*p* < 0.01, ^∗∗∗^*p* < 0.001.

The involvement of the kinase Akt, the endothelial form of NOS (eNOS) and cleaved Notch1 in the G1-induced cardioprotection was evaluated by Western blot analysis. Representative bands and densitometric analyses for these markers are shown in **Figure 5**. In post-ischemic SHR hearts preconditioned with G1, the levels of phosphorylated Akt (**Figure 5A**) and eNOS (**Figure 5B**), and the levels of cleaved Notch1 (**Figure 5C**) were significantly higher, compared to the control counterparts I/R. On the contrary, p-Akt, p-eNOS, and cleaved Notch1 expression levels were significantly reduced by G15 co-treatment (**Figure 5**). The full unedited gels are showed in the Supplementary Figures 2–4, respectively.

#### H9c2 Hypoxia/Reoxygenation (H/R) Protocol

##### G1 induces cardiac cell protection via Notch signaling pathway

**FIGURE 6:**
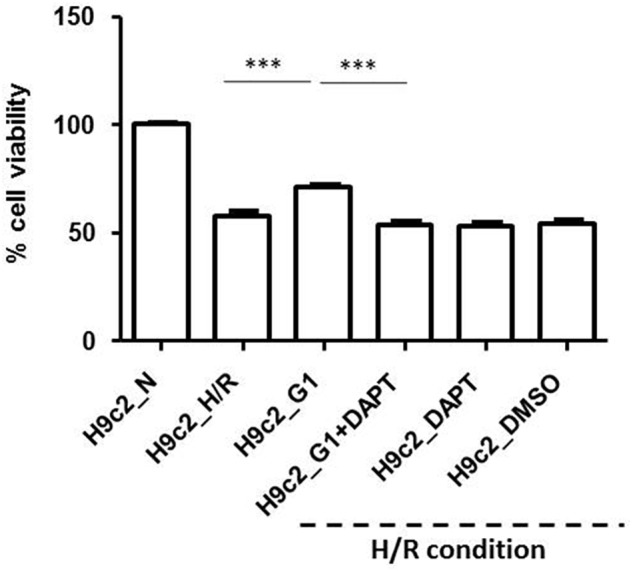
Cell vitality in Normoxia (N) and Hypoxia/Reoxygenation (H/R) conditions. Data are presented as percentage variation with respect to mean value of cell count in normoxia, in control group. Treatment with G1 or G1 in the presence of Notch inhibitor (DAPT) in H/R conditions. Data of control I/R groups with DAPT and DMS are also reported. For acronyms see the text. ^∗∗∗^*p* < 0.001 vs. H9c2_N; ^∗∗∗^*p* < 0.001 H9c2_G1 vs. all other H/R conditions.

In **Figure 6** the effects of H/R in G1 treated cells and after G1 + DAPT pre-treatment compared to normoxic and hypoxic controls (H9c2_N and H9c2_H/R) are reported. DAPT abolished the protective effect induced by G1 pre-treatment against cell death induced by H/R protocol (*p* < 0.001 vs. H9c2_H/R). These results suggest that G1 is an important protective factor during hypoxia able to trigger the Notch1 signaling in cardiac cells.

## Discussion

In this study, we confirm that the cardioprotective role of GPER is Notch1 dependent in normotensive female rats. Importantly, the preconditioning activation of GPER with the selective agonist, G1, significantly reduces I/R injury in the hypertensive female rat heart model. Moreover, in this model G1 is able to activate PI3K/Akt/NOS/MitoKATP channel and Notch1 pathways. The protective effect induced by GPER activation is mediated by Notch1, as also suggested by reduction of cell mortality in the H9c2 model. These results obtained in cardiac cells suggest that the G1 protective effects do not require an endothelial mediation.

The main novel finding of the present study is that the activation of GPER by its selective ligand, G1, protects the heart against I/R injury not only in a normotensive model but also in a model of comorbidity, namely female SHR. In these hypertensive female rats, Notch1 signaling and PI3K/NOS/MitoKATP channel pathways are activated by preconditioning treatment with the GPER agonist G1.

It is well-known that estrogens act through two nuclear receptors: estrogen receptor-alpha (ER-α) or estrogen receptor-beta (ER-β); yet, a third, membrane-bound receptor G protein-coupled estrogen receptor (GPER), has been discovered. GPER has been shown to bind estrogen with high affinity (Zimmerman et al., 2016) and to be localized in the different internal and external cellular localization (Bopassa et al., 2010).

G1 demonstrated high specificity to GPER with little to no binding to other estrogen receptors, either ER-β or ER-α.

Treating acutely animals or perfusing the hearts with 17 beta-estradiol (E2), it has been demonstrated that E2/GPER interaction reduces I/R injury (Deschamps and Murphy, 2009). Moreover, in normotensive condition, the protective effects induced by acute GPER activation induce a remarkable cardioprotection by improving cardiac functional recovery, by reduction the infarct size, and by inhibiting the mPTP opening in isolated rodents hearts exposed to I/R stress (Deschamps and Murphy, 2009; Bopassa et al., 2010; Deschamps et al., 2010; Li et al., 2015; Feng et al., 2017; Menazza et al., 2017). These studies demonstrated that the protective effect of GPER is mediated by the Erk pathway activation, increased superoxide dismutase (SOD), and ATP and decreased the tumor necrosis factor alpha (TNFα) expression level, effects that were also observed in a cardiac cell model subjected to simulated I/R protocols (Li et al., 2015). These results are in agreement with the findings of our study.

As post-conditioning agent, Feng et al. (2017) reported that the cardioprotection of GPER displays via the protection of the mitochondrial structural integrity; moreover, the activation of MEK/Erk signaling leads to the reduction of mitochondrial protein ubiquitination and protection of mitochondrial membrane potential dissipation; these alterations are normally responsible of mitophagy, ROS generation and apoptosis typically of I/R damage (Feng et al., 2017).

Notch signaling pathway seems indispensable for functional activities controlling tissue formation in cardiogenesis (Kratsios et al., 2010). Moreover, it is an important cell–cell communication system and its activation after the damage has been demonstrated in several tissues (Kratsios et al., 2010; Rizzo et al., 2017). It has been reported that Notch signaling plays a regulatory role in adult cardiac damage and in the cardioprotection preserving cardiac function after ischemia. In particular, this Notch activation supports cell survival *via* PI3K induction (Gude et al., 2008). Indeed, in the control of NOS system by Notch1, PI3K/Akt may act as a mediator of eNOS phosphorylation. The blockade of Notch reduced phosphorylation of eNOS and Akt as assessed by western blots in the reperfused hearts (Pei et al., 2013). Several studies report impairment of endothelial Notch signaling in pathological conditions, e.g., heart failure (Pannella et al., 2016), inflammation (Fortini et al., 2017), and dyslipidemia (Briot et al., 2015). Notch signaling can be also impaired by anti-cancer drugs and this may represent an unwanted side effect (Rizzo et al., 2015). The relation of GPER/Notch is demonstrated in other cellular models (Pupo et al., 2016). In particular, we have recently observed that certain non-genomic estrogenic signals are mediated by a functional crosstalk between the Notch signaling pathway and GPER (Pupo et al., 2016). Moreover, we reported that this Notch/GPER crosstalk is involved in proliferative and migratory effects by estrogen, in breast cancer cells and cancer-associated fibroblasts (Pupo et al., 2014).

**FIGURE 7:**
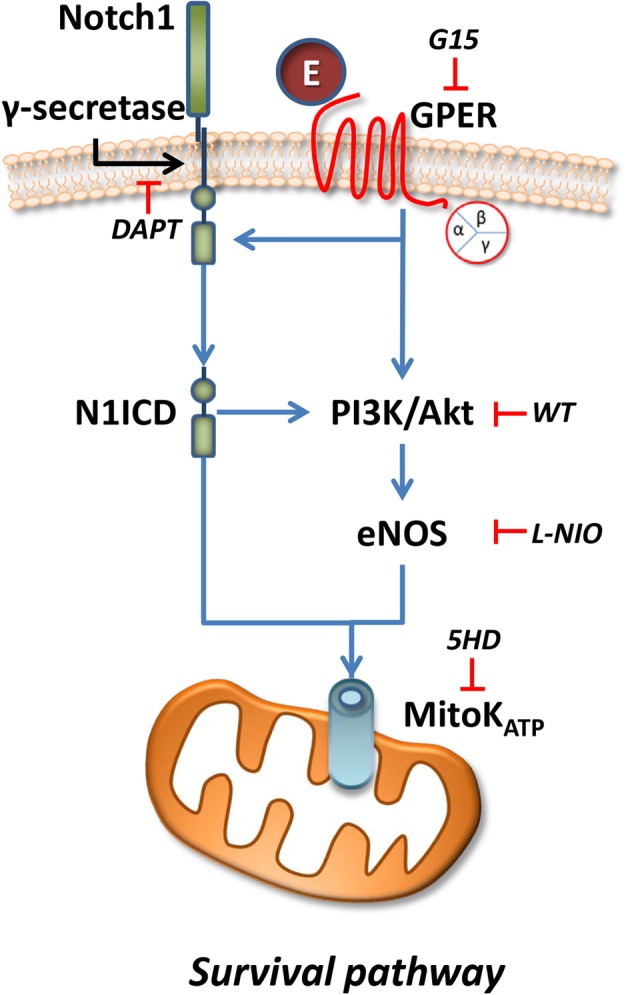
Representative scheme of intracellular pathway involved in the GPER/Notch1 cardioprotection.

Here, for the first time, Notch signaling, nitric oxide, and cell survival pathway are linked to preconditioning protection by GPER activation (**Figure 7**).

Estrogens are hormones regulating physiological and pathological processes in both women and men. Endogenous estrogens affect importantly cardiovascular homeostasis in pre-menopausal women and interfere with the development of hypertension and coronary artery disease. Indeed, the gender differences in cardiovascular risk are surely correlated to hormonal specificity. The estrogens cardioprotective effects are widely reported in several animal and human studies; they are mostly mediated through ERα and/or ERβ, both expressed in the heart and involved in regulating cardioprotection, both in genomic and non-genomic mechanisms (Mendelsohn and Karas, 2005; Wang et al., 2006, 2009; Deschamps et al., 2010).

In addition to the classic ERs, the role exerted by GPER in acute cardioprotection and in the modulation of cardiovascular function is increasingly consolidating (Deschamps et al., 2010).

Our results support the possibility that acute pre-ischemic activation of GPER might protect female hypertensive animals against I/R injury. These results may pave the way for protective approaches in models with risk factors and comorbidities, which are much needed to translate successful animal experiments on cardioprotection beyond that by reperfusion to clinical practice.

## Conclusion

Here, we have shown that GPER plays a pivotal role in mediating preconditioning cardioprotection in both normotensive and hypertensive conditions. This protection requires the involvement of Notch-1 in cardiac cells and is mediated by PI3K/NOS/MitoKATP channels.

## Clinical Perspectives

The pathways here studied may represent novel targets and are particularly intriguing for the advancement in the field of cardioprotection, considering the paucity of studies in female hearts. These results may contribute to better understand the preconditioning GPER protection following ischemia/reperfusion stress in the normotensive and hypertensive heart, an important target tissue for estrogen-mediated signaling. Additionally, the present study may provide more insights for the potential development of a therapy application based on a selective transmembrane estrogen receptor modulator able to induces cardioprotection, without affecting the cancer risk.

## Author Contributions

CP, PR, and TA have devised and coordinated the experiments. CR, SF, GA, MG, EDF, TP, DR, and FF have performed the experiments and analyzed the results. CP and TA have written the first draft. PR and PP revised critically the manuscript. SF, CR, GA, and MG made the figures. CP, PP, PR, MC, MM, and TA finalized the manuscript. All authors approved the final version of the manuscript.

## Conflict of Interest Statement

The authors declare that the research was conducted in the absence of any commercial or financial relationships that could be construed as a potential conflict of interest.
