# Supplementary figures and images for: Notch1 Mediates Preconditioning Protection Induced by GPER in Normotensive and Hypertensive Female Rat Hearts
Source: Front Physiol. 2018 May 15;9:521. doi: 10.3389/fphys.2018.00521 (PMC5962667; doi:10.3389/fphys.2018.00521)

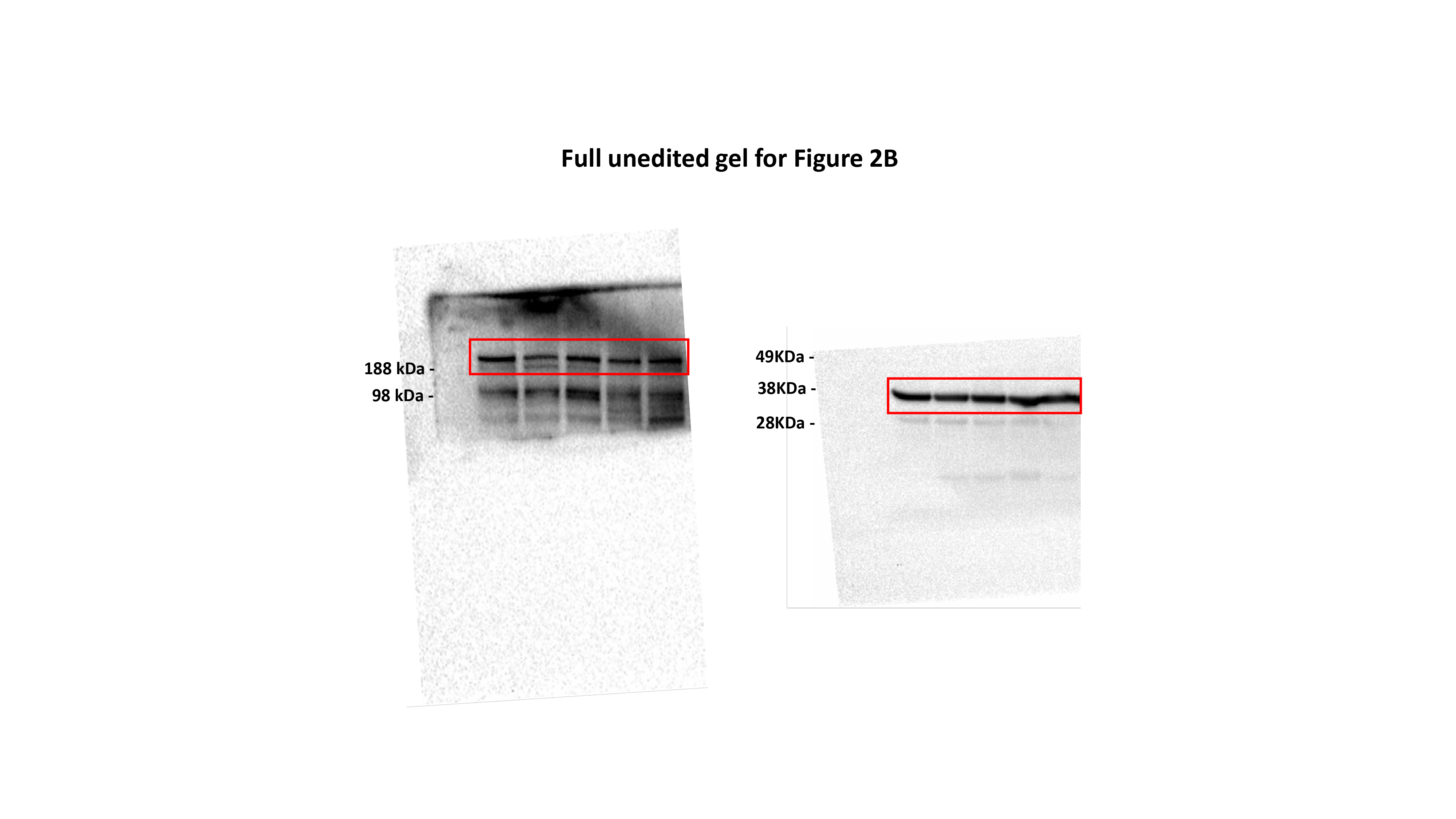

Supplement: Supplementary file 1 [file Image_1.TIF]

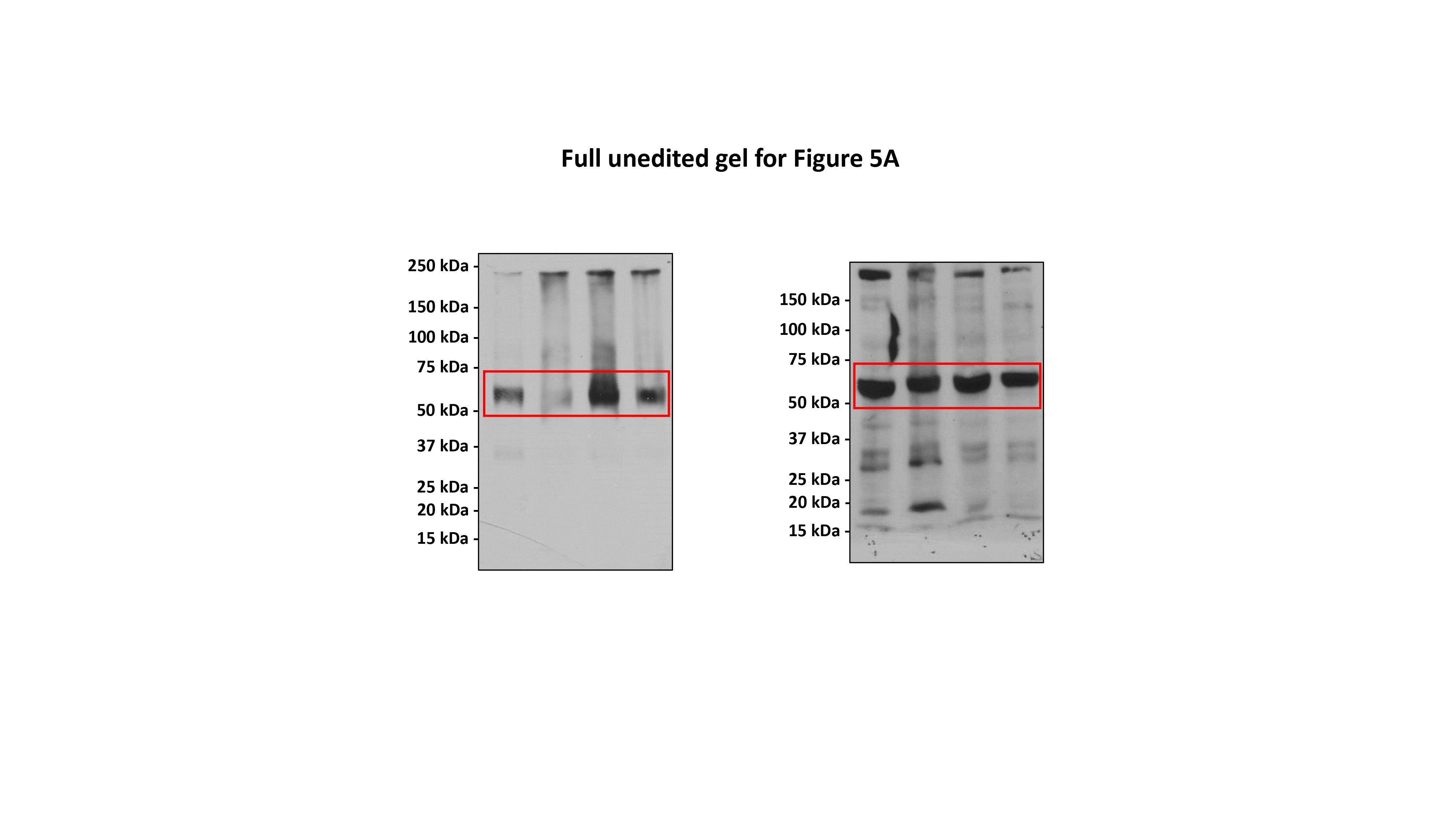

Supplement: Supplementary file 2 [file Image_2.TIF]

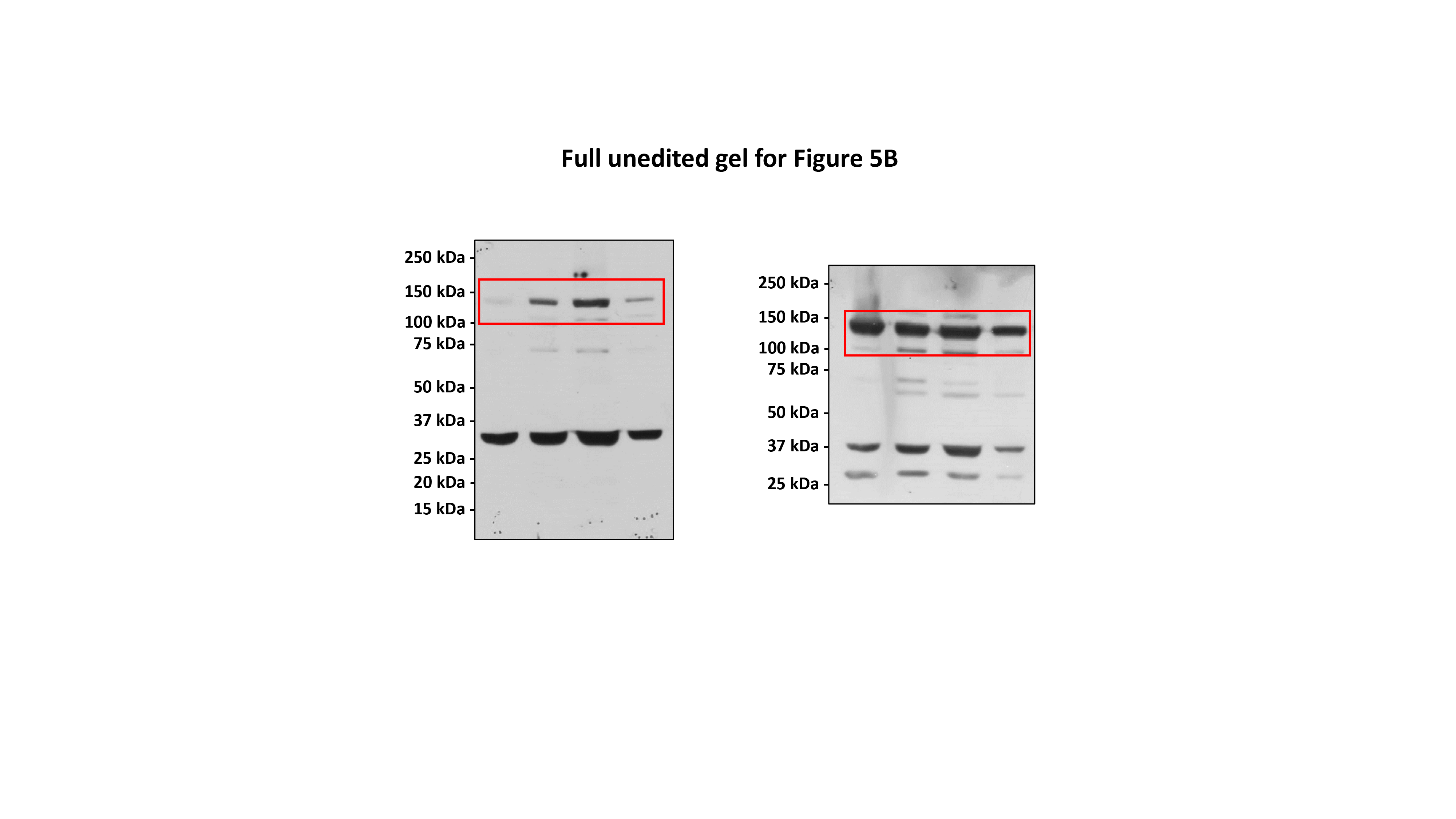

Supplement: Supplementary file 3 [file Image_3.TIF]

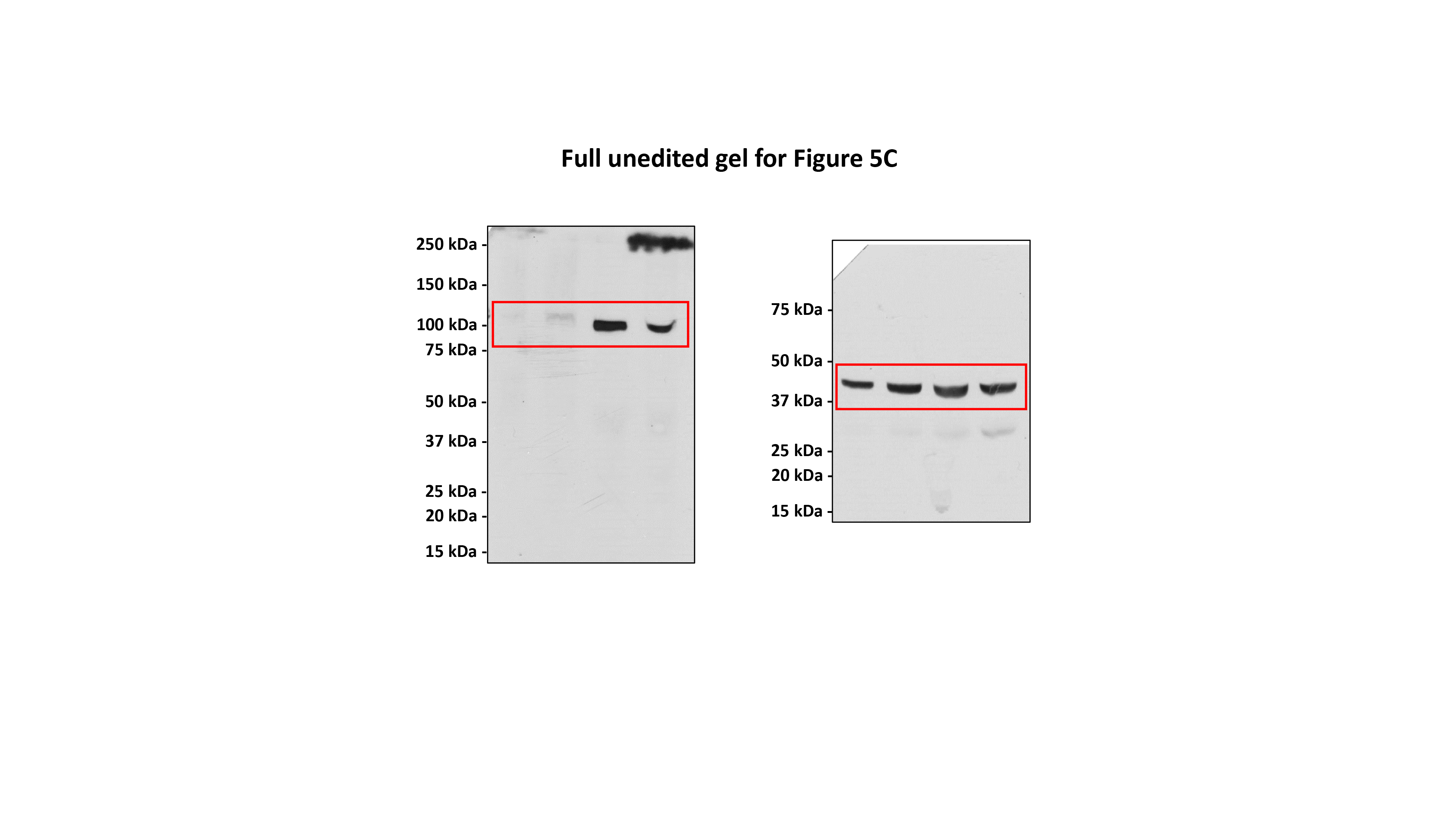

Supplement: Supplementary file 4 [file Image_4.TIF]
